# Iodized Glycerine in Skin Diseases

**Published:** 1860-07

**Authors:** 


### IODIZED GLYCERINE IN SKIN DISEASES.

This solution is prepared after the following formula: ℞. Potassii iodidi, et iodini, each ℨ i.; glycerine, f ℨ ij. Add the iodide of potassium to the glycerine, and when a solution is effected, add the iodine. A few minutes' agitation will cause a perfect dissolution.

This solution has the great advantage over alcoholic solutions of not drying; in consequence the surfaces remain supple and the absorption and action of the iodine is much prolonged. It should be applied to the affected part, and covered with gutta-percha paper, to prevent evaporation, and increase the perspiration of the part. It is left untouched for twenty-four hours, and the degree of re-action regulates its further application. The application of water will readily remove all traces of the solution. This solution occasions pain, which varies in intensity and duration according to the state of the diseased part and the sensitiveness of the patient. There has, however, never been any general inconvenience. On removing the application, the healthy skin has become brown and the diseased parts paler than before. On ulcerating surfaces, no trace of iodine will be found two hours after its application. Sometimes its action has been so powerful as to produce phlyctene.

The results of Dr. Richter's experiments are, that this solution acts as a caustic; that it has really a heroic action in cases of lupus; that its efficacy is remarkable in non-vascular goitre, scrofulous ulcers, constitutional syphilitic ulcers—doubtful in primitive chancres and eczema, and useful in psoriasis.—*Wener Med. Wochens-Schrift.*

